# GPRI: Dr. Asmaa Abdel Sameea Mahmoud

**DOI:** 10.1038/s41390-022-02324-0

**Published:** 2022-10-04

**Authors:** Asmaa Abdel Sameea Mahmoud

**Affiliations:** Menoufia University, Pediatric Department, Faculty of Medicine, Shebin El Kom, Egypt

I would like to thank the editor for allowing me to share the seventeen years of my past life with the readers of this prestigious and distinguished magazine. I grew up in the city of Berkit El-Sabaa, Menoufia Governorate, Egypt.
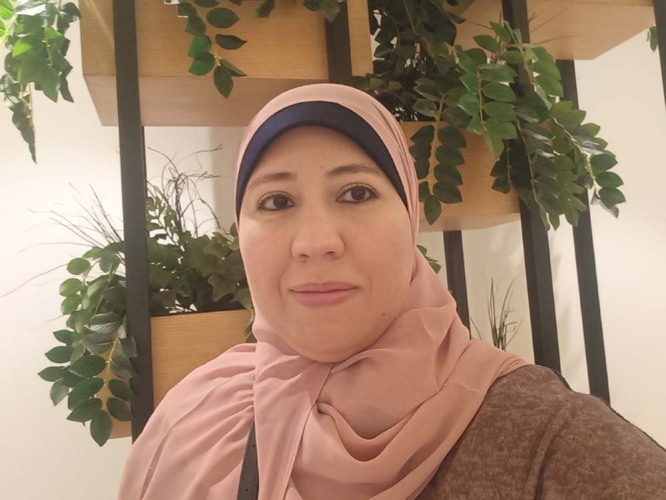
**My personal Image**

I joined the Faculty of Medicine, Menoufia University, and specialized in pediatrics, especially hematology and oncology. I spent a great deal of my life among these patients, and the first research was done on fungal infections among oncology patients with febrile neutropenia.

I was fortunate and happy to work with Prof. Dr. Farida Hussein Al-Rashidi, Prof. Dr. Fady Mohamed Elgendy, Prof. Dr. Ahmed Anwar Khattab and their encouragement to complete this research and add the new in the treatment of fungal infection among these patients, as this research was the first research in Egypt in this field in this period. I would like to thank Prof. Dr. Ghada Mohamed Elmashad for giving her my first research after receiving my MD degree.

After that, I completed the scientific research with the encouragement of my older sister Prof. Dr. Eman Abdel Sameea Mahmoud and my colleagues (Dr. Nagwan Yossery Saleh, Dr. Sameh Abdallah Abd El-Naby, Dr. Nahla Mohamed Said and Dr. Faten Ezzelarab Younis). To all who read this synopsis, we want to enjoy a stable psychological state in order to complete the process of practical and scientific life. The spirit of cooperation and perseverance is the basis of success in everything, and we should set a goal and strive to achieve it.

